# MERTK-Mediated LC3-Associated Phagocytosis (LAP) of Apoptotic Substrates in Blood-Separated Tissues: Retina, Testis, Ovarian Follicles

**DOI:** 10.3390/cells10061443

**Published:** 2021-06-09

**Authors:** Marina G. Yefimova, Celia Ravel, Antoine D. Rolland, Nicolas Bourmeyster, Bernard Jégou

**Affiliations:** 1 Sechenov Institute of Evolutionary Physiology and Biochemistry, Russian Academy of Sciences, 44, Prosp. Maurice Thorez, 194233 St-Petersburg, Russia; 2 Département de Gynecologie Obstetrique et Reproduction Humaine—CECOS, CHU de Rennes, 16, Boulevard de Bulgarie, 35000 Rennes, France; Celia.RAVEL@chu-rennes.fr; 3 Univ Rennes, Inserm, EHESP, Irset (Institut de Recherche en Santé, Environnement et Travail)-UMR_S 1085, F-35000 Rennes, France; antoine.rolland@univ-rennes1.fr (A.D.R.); bernard.jegou@inserm.fr (B.J.); 4 Laboratoire Signalisation et Transports Ioniques Membranaires, CNRS ERL 7003, Université de Poitiers, 1 rue Georges Bonnet, F-86022 Poitiers, France; nicolas.bourmeyster@univ-poitiers.fr; 5 Service de Biochimie, CHU de Poitiers, 1, rue de la Milétrie, 86021 Poitiers, France

**Keywords:** Mer tyrosine kinase, phagocytosis, autophagy, LAP, retina, testis, ovaries

## Abstract

Timely and efficient elimination of apoptotic substrates, continuously produced during one’s lifespan, is a vital need for all tissues of the body. This task is achieved by cells endowed with phagocytic activity. In blood-separated tissues such as the retina, the testis and the ovaries, the resident cells of epithelial origin as retinal pigmented epithelial cells (RPE), testis Sertoli cells and ovarian granulosa cells (GC) provide phagocytic cleaning of apoptotic cells and cell membranes. Disruption of this process leads to functional ablation as blindness in the retina and compromised fertility in males and females. To ensure the efficient elimination of apoptotic substrates, RPE, Sertoli cells and GC combine various mechanisms allowing maintenance of tissue homeostasis and avoiding acute inflammation, tissue disorganization and functional ablation. In tight cooperation with other phagocytosis receptors, MERTK—a member of the TAM family of receptor tyrosine kinases (RTK)—plays a pivotal role in apoptotic substrate cleaning from the retina, the testis and the ovaries through unconventional autophagy-assisted phagocytosis process LAP (LC3-associated phagocytosis). In this review, we focus on the interplay between TAM RTKs, autophagy-related proteins, LAP, and Toll-like receptors (TLR), as well as the regulatory mechanisms allowing these components to sustain tissue homeostasis and prevent functional ablation of the retina, the testis and the ovaries.

## 1. Phagocytosis, General Information, and Types of Phagocyte Cells

Every day, around 20–50 billion cells die by apoptosis and have to be eliminated from the body in order to maintain homeostasis and to avoid the autoimmune response against intracellular antigens [1,2]. Therefore, once formed, apoptotic cells and cell debris are immediately eliminated by cells endowed with phagocytic activity. Over the last few years, important progress has been made on the deep understanding of the different steps and molecular support of phagocytosis of apoptotic substrates. Several excellent reviews are available to date [3,4]; therefore, here we will only briefly touch upon the general information regarding phagocytosis process per se, and then focus on phagocytosis mediated by c-mer proto-oncogene tyrosine kinase (MERTK) in blood-separated tissues.

In the classical definition, phagocytosis is a complex, tightly regulated process, which consists of three principal steps: the recognition and binding of the substrate (step 1), followed by its internalization (step 2), and completion of the process by its degradation by lysosomes (step 3) [5]. A plethora of different receptors on phagocyte plasma membranes recognize particular molecular markers on the surface of apoptotic substrates, thereby allowing their binding and the realization of step 1 of phagocytosis. Nevertheless, only a limited number of these receptors are responsible for target ingestion [6], which corresponds to step 2 of phagocytosis. The ingestion is not automatically triggered by the initial binding of the particle to the phagocyte; it requires sequential interaction of phagocyte receptors with ligands on the remainder of the particle’s surface [7]. Moreover, ingested substrate cannot be directly addressed for degradation (step 3), but can accumulate in cell cytoplasms [8].

Depending on their origin (hematopoietic or not), the phagocytes are termed as professional or non-professional, respectively [9]. In a normal situation, freely circulating professional phagocytes from the bloodstream cannot infiltrate the blood-separated tissues. In this case, the resident cells of epithelial origin act as non-professional phagocytes, providing the clearance of tissue-specific substrates. This contributes to tissue homeostasis; therefore, such a type of phagocytosis is termed as homeostatic phagocytosis [10].

## 2. Non-Professional Phagocytes from Blood-Separated Tissues and Their Substrates

Among blood-separated tissues, the retina, the testis and the ovaries manifest the highest rate of the production of apoptotic substrates, therefore requiring efficient machinery for their removal.

Thus, in the eye, numerous portions of the retinal photoreceptor cell membrane (packaged into discs) are shed daily from living photoreceptors and ingested by retinal pigmented epithelium (RPE) cells [5,11]. In rats, each RPE cell contacts about 250–300 photoreceptor outer segments (POS), and each RPE ingests some 25,000–30,000 disks every day [12]. It has been estimated that in 80 years old humans, each single RPE cell will have engulfed some 108 shed POS [13].

In the mammalian testis, germ cell death is a prominent event during normal spermatogenesis. As estimated, it results in the loss of up to 75% of the potential number of mature spermatozoa [14,15]. Furthermore, 250 × 10^6^ residual bodies (RB), which are the fragments of apoptotic membranes detached from elongated spermatids [16], are produced daily by the mammalian testis. Both apoptotic germ cells and RB are ingested and destroyed by the somatic Sertoli cells [17,18], which are “nurse” cells of the testis, forming a part of a seminiferous tubule and supporting the process of spermatogenesis [19].

The ovary is also an extremely dynamic organ, in which 99% of ovarian germ cells undergo apoptosis through follicular atresia [20]. An ovarian follicle, which is a basic unit of female reproductive biology, consists of single oocyte surrounded by a layer of GC and enclosed in basal lamina. Apoptotic death affects oocyte at primordial, primary and small preantral phases of follicle maturation. Elimination of apoptotic oocytes relies upon phagocytic activity of GC [21], which share striking similarities with testis Sertoli cells with respect to their origin and function. According to the standard model of sex determination, both the Sertoli cells and the GC directly stem from the supporting cell precursors of the bipotential gonad [22].

## 3. Autophagy: A Lysosome-Related Degradation Pathway Sharing Similarities with Phagocytosis

Degradation of ingested substrates by phagocyte cells relies upon the hydrolytic activity of lysosomes. Lysosomes are also involved in the degradation of intracellular components through autophagy machinery. Autophagy was classically defined as the starvation survival mechanism, endowing cells with the ability to auto digest. This mechanism allows the degradation and reuse of the portions of cell cytoplasm in support of vital functions [23,24]. In the past, autophagy was considered a non-selective process by which randomly enclosed cargo was degraded in bulk. However, recent findings suggest that autophagy is a highly selective process, specifically targeting cellular organelles and cytoplasmic components, such as mitochondria, ribosomes, peroxisomes, lipid droplets, misfolded proteins, and intracellular pathogens [25]. Autophagy participates in a broad spectrum of biological processes, including ageing, development, degenerative diseases and cancer, being triggered by variety of stimuli both in vitro and in vivo [26]. Autophagy is heralded by the formation within the cytosol of crescent-shaped nascent autophagic isolation membranes (phagophores) that corral cytoplasmic targets into short-lived double membrane autophagosomes. After maturation and fusion with a lysosome, the captured cargo is exposed to the degradative lysosomal enzymes [27] that decompose large molecules into their basic units. Autophagy has a nearly unlimited degradative capacity. It can target proteins, lipids, both DNA and RNA as well as entire organelles, thereby providing new pools of amino acids, fatty acids and nucleosides for anabolic processes [28]. To date, 37 autophagy-related genes (ATG) have been identified in yeast, with orthologues well conserved throughout eukaryotes [29].

A range of signaling processes converge on two protein complexes to initiate autophagy: the ULK1 (Unc-51 like autophagy activating kinase 1) protein kinase complex and the PI3KC3-C1 lipid kinase complex [30]. The ULK1–Atg13–FIP200 (FAK family kinase-interacting protein of 200 kDa) complex regulates autophagosome synthesis downstream of the mTOR (mechanistic target of rapamycin) signalling pathways. The PI3KC3 complex, comprising hVps34, Beclin-1 and hVps15, also regulates autophagosome synthesis, possibly downstream of the mTOR-independent pathways. A key regulatory mechanism of autophagy is that Beclin 1 is complexed with Bcl-2 family proteins; the dissociation of Bcl-2 from the Beclin 1-hVPS34 complex activates autophagy [31,32]. The dissociation of the Bcl-2–Beclin 1 complex is regulated by kinases, tumor suppressors [27], as well as key adapter proteins in innate immunity signaling downstream of TLRs, such as MyD88 and TRIF [33]. Several Beclin-1 interactors, such as Atg14L, UVRAG (UV radiation resistance-associated gene protein), Bif-1 (Bax-interacting factor 1) and Ambra1 (Autophagy and Beclin 1 Regulator 1), or Rab5 (Ras-related protein 5) interacting with hVps34, positively modulate this process. Two ubiquitin-like conjugation systems involving Atg proteins function in the elongation of phagophores. The Atg5–Atg12 conjugation involves Atg7 and Atg10, whereas the LC3–PE (Microtubule-associated protein 1A/1B-light chain 3—Phosphatidylethanolamine) conjugation involves Atg7 and Atg3. The Atg5–Atg12 conjugate forms a complex with Atg16, Atg5–Atg12·Atg16, which demonstrates E3-like (ubiquitin ligase) activity towards LC3–PE conjugation (LC3-II). LC3-II is a specific autophagy marker currently used to monitor autophagy [34].

## 4. Autophagy in Non-Professional Phagocytes from Blood-Separated Tissues

Autophagy degradation machinery plays a significant role in homeostasis maintenance in RPE, GS and Sertoli cells. A high basal autophagy level is maintained in RPE cells, and the light exposure causes an autophagic response in mice [35,36,37,38,39]. Several experimental studies indicate that compromised autophagy in RPE cells is associated with early signs of retinal degeneration [40,41,42], and impairing autophagy leads to RPE apoptosis [42]. RPE from patients with late stages of age-related macular degeneration (ARMD) manifest decreased levels of autophagy proteins, suggesting their role for normal ocular physiology [36]. Autophagy was shown to protect RPE cells from the toxic effects of oxygen peroxide and ethanol treatments, while inhibition of autophagy abolished the protective effect [40,41].

A high basal level of autophagy has also been detected in Sertoli cells [43,44], wherein it supports several basal physiological processes. Thus, autophagy is implicated in the testosterone-regulated clearance of androgen-binding proteins (ABP) [45]. Testosteron-induced upregulation of ABP expression is thought to be related to the suppression of autophagic degradation of ABP. The knocking down of autophagy genes in Sertoli cells increases germ cell apoptosis, reduced spermatozoa count and abnormal spermiation [46]. A protective effect of autophagy for Sertoli cell survival has been demonstrated in acute ethanol-induced exposure in rodents [47,48]. In contrast to germ cells, which undergo apoptosis after ethanol exposure, Sertoli cells resist apoptosis through the upregulation of autophagy at the spermiation stages of the spermatogenic cycle [47,48]. Moreover, following acute ethanol exposure, numerous apoptotic germ cells and spermatozoa are found within the cytoplasm of Sertoli cells, indicating the role of autophagy proteins in the cleaning of seminiferous tubules from apoptotic substrates [47,48].

The expression of autophagy proteins in GC has been detected by numerous teams [49]. Experimental evidence indicates that autophagy is involved in the processes of follicular growth and atresia [49]. Mice with *Atg7* gene knockdown typically produce small litters and become sterile over time [50]. The ovaries of *Atg7*-deficient mice always contain fewer germ cells and primordial follicles; the latter are often structurally altered and non-functional. In vitro autophagy in GC is modulated by gonadotropin treatment [51,52], and the exposure of GC to oxidized low-density lipoprotein [53]. In vivo, the activation of mTOR mediated by AKT suppresses GC autophagy during follicular atresia in rats [54], suggesting the crucial role of autophagy for female gametogenesis.

## 5. Non-Canonical Roles of Autophagy

Recent evidence has established that a variety of non-canonical functions of autophagy proteins are mechanistically and functionally distinct from autophagy [55]. Several autophagy proteins from canonical autophagy machinery are used in separate processes, such as LANDO (LC3-associated endocytosis pathway), which is involved in the recycling of amyloid receptors and in the clearance of amyloid aggregates by microglia in a murine model of Alzheimer’s Disease (AD) [56]; CUPS (compartment for unconventional protein secretion) for unconventional secretion of proteins without signal peptides as acyl-CoA binding protein 1 and interleukin-1β [57,58,59,60,61]; DRibbles (Defective Ribosomal Products-Containing Autophagosome-Rich Blebs)-for secretion of exosome-like fractions modified by autophagosomes [62,63,64]; and LAP (LC3-associated phagocytosis) - a hybrid process, in which autophagy proteins support phagocytosis degradation machinery [55] (see below, § 9).

## 6. Ablation of Phagocytic Activity by Resident Non-Professional Phagocytes Causes Blindness in the Retina and Compromised Fertility in the Testis and the Ovaries

**Figure 1:**
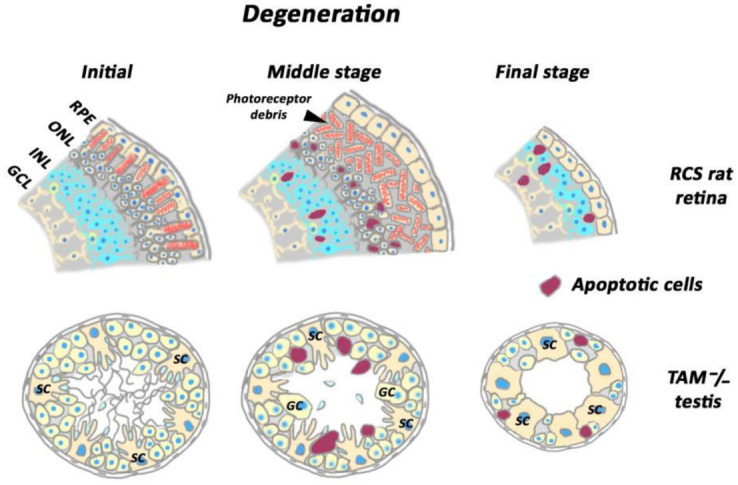
Degenerative changes in RCS rat retina and in the testis of triple mutant TAM−/− mice. Abbreviations: GCL—ganglion cell layer, INL—inner nuclear layer, ONL—outer nuclear layer, RPE—retinal pigmented epithelium, SC—Sertoli cells, GC—giant cell.

To date, the notion of the importance of homeostatic phagocytosis for functional consistency of blood-separated tissues is widely accepted. Ablation of phagocytic ability by resident non-professional phagocytes has been shown to cause dramatic outcomes for blood-separated tissues, including the loss of their specific functions and their degeneration. This was first demonstrated in a genetic model of retinal degeneration in Royal College of Surgeons (RCS) rats. This naturally occurring rat strain was described in the first half of the 20th century [65]. Subsequent long-term study of this model showed that an irreversible blindness in RCS rats was caused by the inability of retinal pigmented epithelium to ingest the tips of POS shed from photoreceptor cells in vivo and in vitro [66]. The pattern of degeneration in RCS rats has been thoroughly documented using histological and electron-microscopy techniques (Figure 1). Thus, it was established that by 3 weeks of age, photoreceptor outer segments showed evidence of disruption with the development of an apical debris zone. By 4 weeks, pyknotic photoreceptor nuclei were observed in the outer nuclear layer. Between 7 and 12 postnatal weeks, the outer nuclear layer was reduced to a single layer of photoreceptor cell bodies and the debris zone occupied the former outer segment area [67,68]. TUNEL staining indicated that apoptosis was the dominant mechanism of photoreceptor degeneration in the RCS [69]. Further deterioration of the debris zone allows the inner nuclear layer to come into close contact with RPE by 24 weeks of age. Secondary neurodegenerative changes include the loss of retinal ganglion cells, withdrawal of bipolar cell axons, Müller cell gliosis, neuronal sprouting of horizontal cells and microglial activation [67,68,70,71]. To date, the RCS rat provides a model to study various cellular interactions and triggering mechanisms leading to this secondary photoreceptor cell death. There is not an uniform opinion on the cause of photoreceptor cell death. Thus, the debris zone acting as a diffusion barrier to metabolite, disruption of the interphotoreceptor matrix, photoreceptor hypoxia due to diminished oxygen diffusion, and gradual accumulation of toxic iron deposits in the layer of photoreceptor debris have been proposed as possible explanations [72,73]. The latter explanation concerns iron ions released from the iron-transport protein transferrin, which undergoes a non-specific degradation in the debris zone. In normal retina, transferrin is secreted by RPE cells in order to deliver this vital element to photoreceptor cells [73,74]. The degradation of transferrin results in the liberation of iron ions which accumulate in debris zone. This triggers oxidative stress [75], which contributes to the apoptotis of photoreceptors [69,76]. Intravitreal injections or non-viral delivery of transferrin in its iron-free form (apotransferrin) to chelate unbound iron, or antioxidant biomolecules, delay retinal degeneration in RCS rats, supporting the consistency of such a scenario [75,76,77].

In 2000, two independent research groups concurrently identified a single gene defect, the *Mer* tyrosine kinase (*Mertk*) [78,79,80]. The deletion in the *Mertk* cDNA [79], resulting from a large genomic deletion [78], led to the production of non-functional 20-amino-acid Mertk proteins in RPE cells. The latter failed to internalize POS. Subsequent studies demonstrated that transgenic (*Mertk* *KD*) mice developed the same phenotype as RCS rats [81], resembling the symptoms of retinitis pigmentosa patients. Mutations of *Mertk* in humans were shown to be associated with retinitis pigmentosa and other ocular pathologies [82,83]. Further studies demonstrated that the subretinal injection of a recombinant replication-deficient adenovirus encoding rat *Mertk* to the eyes of young RCS rats caused the substantial sparing of photoreceptors, preservation of the outer segment structure, and correction of the RPE phagocytosis defect in areas surrounding the injection site [72]. Electrophysiological assessment of animals 30 days after injection revealed the increased sensitivity of treated eyes to low-intensity light. These results provided definitive evidence that the mutation of *Mertk* underlies the RCS retinal dystrophy phenotype, and that the phenotype can be corrected by the treatment of juvenile animals [72].

In parallel with ocular studies throughout the last decade of the 20th century, another research team specialized in reproduction biology generated and analyzed null mutations in the mouse genes encoding three structurally related receptors with tyrosine kinase activity: *Tyro 3, Axl,* and *Mer* (TAM family) [84]. These mice presented increased apoptosis and cellular degeneration in a variety of adult tissues; however, only two adult tissues were found to manifest complete loss of specific function, namely the testis and the retina. *TAM−/−* triple mutants were blind due to degeneration of photoreceptor cells; the adult males were sterile, never siring offspring. The testes of adult mice were one-third of the size of those of wild type (WT) ones, seminiferous tubules presented perturbed cellular organization, and epididymis was invariably devoid of sperm (Figure 1). Further study revealed no evidence of impaired proliferation of spermatogonia in adult *TAM−/−* mice, but detected increased germ cell apoptosis and an emergence of giant cells in seminiferous epithelium. Consistent with the loss of germ cells, the seminiferous tubule diameter was significantly decreased in *TAM−/−* mice at 15 weeks of age and older, reflecting a decrease in total germ cell complement. Extensive study of seminiferous tubule sections allowed the conclusion that, in *TAM−/−* mice, the germ cells were progressively lost from elongated spermatids to spermatocytes, and finally, the observation of spermatogonia as the mice aged. Furthermore, young adult *TAM−/−* mice exhibited oligo-astheno-teratozoospermia and various morphological malformations of sperm cells [85,86]. *TAM* double mutants were also compromised with respect to spermatogenesis, although to a lesser extent; among double mutant mice, the *Tyro3−/−Axl−/−Mer+/+* phenotype was less severe with respect to fertility and testicular size, and all single mutant males appeared normal. Of note is that *Mer−/−* mutants were blind [84]. Maturation and functions of testosterone-producing Leydig cells were not apparently affected in *TAM−/−* mice, as well as serum testosterone levels [85,86]. Subsequent in situ hybridization (ISH) study revealed that mRNA for TAM receptors was confined to the Sertoli cells during postnatal development [84]. Because no TAM expression was detected in germ cells, it was concluded that impaired spermatogenesis should not be germ cell autonomous, but relies upon a functional disability of Sertoli cells which are critical for normal spermatogenesis [87].

Though the authors of this original paper [84] did not discuss spermatogenesis failure as a consequence of phagocytosis defected by Sertoli cells induced by TAM mutation, subsequent studies by other research teams shed light on this causative link [85,86]. Using primary culture of Sertoli cells isolated from the testis of TAM mutant mice, the rate of phagocytosis of apoptotic germ cells was assessed for single, double or triple mutant. The data obtained indicated that Sertoli cells lacking Mertk showed a 35% reduction in phagocytosis rate, whereas cells lacking Tyro3 or Axl exhibited no reduction in comparison to WT control. Sertoli cells from triple TAM mutants showed a dramatic decrease in phagocytosis rate of 7.6-fold when compared to the wild type Sertoli cells [85,86]. Collectively, the data presented the first experimental evidence for the importance of TAM receptors for phagocytosis function by Sertoli cells in vitro.

The defect of Sertoli cell phagocytosis is currently considered a major cause of the impaired spermatogenesis in TAM mutant mice [85,86,87,88,89]. One explanation for how the timely removal of apoptotic cells and residual bodies contributes to spermatogenesis is the prevention of leaking out noxious contents and poisoning healthy cells [88]. Another explanation concerns energy balance in seminiferous tubules. As evidenced, the apoptotic spermatogenic cells and residual bodies could be used to produce energy by Sertoli cells after the phagocytosis of them [90]. The net output of energy by Sertoli cells is important not only to spermatogenesis, but also to germ cell movement [91]. Impaired Sertoli cell phagocytosis may result in a decrease in the energy production by Sertoli cells as well.

Studies on *TAM−/−* triple mutant mice also revealed reduced fertility in females [84], but this finding remained unnoticed for years. To our knowledge, there is no litterature data depicting ovarian histology in *TAM−/−* mutant mice. The idea of the involvement of GC in removing apoptotic oocytes emerged from transcriptomic analysis of bovine GC from small antral follicles undergoing atresia. Indeed, a 4-fold increase of Mertk expression was detected in GC, surrounding small antral follicles [92]. A subsequent study confirmed the role of GC in the removal of apoptotic oocytes through MERTK-mediated phagocytosis [21]. Further investigation is needed to clarify a possible scenario leading to compromised fertility in females carrying mutation in the *MERTK* gene, and its relationship with phagocytosis by GC.

## 7. MERTK Is a Member of the TAM Family of Receptor Tyrosine Kinases

The TAM family of receptor tyrosine kinases (RTKs) includes Tyro3, Axl and Mer members which share similarities in structure and function. TAM receptors are expressed in many cell types, but the pattern of individual receptor expression varies. Mer is highly expressed in monocytes/macrophages, testis, ovaries and epithelial cells (including the RPE) [93,94,95].

In recent years, several signaling functions of TAM receptors have been described, such as stimulation of cell growth and proliferation, inhibition of apoptosis, stimulation of hemostasis, modulation of inflammation and phagocytosis of apoptotic substrates by professional [96,97] and non-professional phagocytes [98]. In professional phagocytes, the requirement of each member of the TAM family depends on the type of phagocyte: in macrophages, Mer is essential for rapid ingestion of apoptotic cells, though Axl and Tyro3 are partly involved. In dendritic cells, Axl and Tyro3 appear to function, and Mer has no role [97,99]. In microglia, Axl and Mer, but not Tyro3, are implicated in apoptotic substrate phagocytosis [100].

TAM are transmembrane proteins composed of an extracellular region, containing two immunoglobulin-like (IG-like) domains, linked to two fibronectin type III repeats and a cytoplasmic region containing a protein tyrosine kinase domain [101]. TAM receptors may signal either in response to ligand activation or by formation of homodimers or heterodimers with each other or other receptor tyrosine kinases to signal independently of their ligands [102,103,104]. Ligand binding to the extracellular domain induces receptor dimerization and the subsequent trans-autophosphorylation of tyrosine residues within the cytoplasmic domain. This results in the subsequent activation of the complex cascade of signaling molecules [105]. To date, at least five different molecules, namely Growth Arrest Specific protein 6 (GAS6), Protein S (PROS1) [106], tubby, Tulp1 [107] and galectin 3 (Gal3) [108], have been described as TAM ligands.

Among TAM ligands, GAS6 and PROS1 recognize the apoptotic marker phosphatidylserine (PS) exposed on the outer membrane leaflet of the apoptotic membrane [109]. TAM ligands bridge apoptotic substrate to TAM receptors on the plasma membrane of the phagocyte. Almost all of bridging molecules are expressed by blood-separated retina, testis and ovaries, suggesting the importance of TAM signaling in these tissues [84,109,110,111,112,113,114,115,116,117,118,119]. Moreover, data from animal models indicate that, in the testis, such multiligand bridging provides the phagocytic clearance of apoptotic substrates even when one and/or two of the bridging molecules are knocked down (*Gas6−/−* mice [120], double KO *Gas6/ProS−/−* mice [121], *Gal3−/−*mice [122], and *Tub* null mice [123]). In the case of double KO mice, such functional compensation seems to provide an advantage for the Sertoli cell (and probably for GC) over RPE cells which also use TAM RTKs to maintain tissue homeostasis.

## 8. MERTK Cooperates with Other Phagocytosis Receptors to Provide the Ingestion of Apoptotic Substrates

Phagocytosis of apoptotic substrates by RPE, testis Sertoli cells and ovarian GC is associated with the activation of MERTK [21,44,124,125]. Nevertheless, studies from TAM mutant mice showed that homeostatic phagocytosis machineries from testis and ovaries were able to maintain phagocytic function even when some of the receptors were knocked down [84]. On the contrary, phagocytosis by RPE cells was completely arrested in *Mertk −/−* mice [81], suggesting the pivotal role of this receptor for maintaining ocular homeostasis and visual function.

Phagocytosis by RPE cells has been extensively studied by numerous teams, so the main events allowing POS to be ingested by RPE cells have been established. Though having a primordial role in POS phagocytosis, Mertk cooperates with Tyro3 [126] and other surface-bound receptors, which provide tight attachment of POS to the phagocyte plasma membrane and trigger downstream signaling. POS detached from photoreceptor cells are “apoptotic-like” substrates exposing PS, which binds to the cluster of differentiation 36 (CD36) on apical membranes of RPE cells [127]. Furthermore, the αVβ5 integrin receptor also binds POS by bridging milk globular factor E-8 (MGF-E8) protein secreted by RPE cells. The binding of POS to αVβ5 integrin receptors stimulates both MERTK (through αVβ5 integrin-associated focal adhesion kinase (FAK) [128]) and Ras-related C3 botulinum toxin substrate 1 (RAC1)-GTPase [129], leading to actin recruitment to the phagocytic cup. Moreover, Mertk is also activated after binding with GAS6 and/or PROS1 bound to POS. Activation of RAC1-GTPase downstream of Mertk phosphorylation has also been demonstrated for testis Sertoli cells during phagocytosis of apoptotic substrates [44]. In RPE, phosphorylation of Mertk generates docking sites for phosphatidylinositol-4,5-bisphosphate 3-kinase (PI3) kinases [130], which is required for the recruitment of F-actin to the phagocytic cup [127]. Besides, Mertk signaling induces the recruitment of myosin II to close the phagocytic cup and to move apoptotic substrates inside phagocytes as demonstrated for RPE, GC and Sertoli cells [21,44,124].

To date, no information on multireceptor complexes providing membrane remodeling and ingestion of apoptotic substrates in testis and ovaries is available. Nevertheless, the scavenger receptor CD36 [131] and integrins and bridging molecules GAS6, PROS1 and MGF-E8 are expressed in testis and ovaries [132,133,134,135,136]. Moreover, the translocation of the CD36 receptor across the Sertoli cell towards the sites of accumulation of RBs and apoptotic germ cells has been shown both in vitro and in vivo [131]. CD36 is highly expressed in GC [137], and *CD36* null mice display ovarian morphology resembling those seen in polycystic ovarian syndrome (PCOS), in which there is an abnormally increased number of primary follicles which remain preovulatory [138]. Furthermore, the variations of FAK phosphorylation and RAC1-GTPase driven actin re-arrangement depending on various stages of seminiferous epithelium and ovarian cycles have been detected as well [139,140,141,142]. Further studies are needed to identify the member of phagocytosis machinery providing the cleaning of apoptotic substrates in testes and ovaries.

## 9. MERTK-Mediated Phagocytosis in Blood-Separated Tissues Is an Autophagy-Assisted Process Termed LAP

In 2013, two independent studies demonstrated that homeostatic phagocytosis machinery in the retina and in the testis required the involvement of autophagy-related proteins [44,143]. These data were further supported by [18,21], who confirmed the recruitment of autophagy-related proteins for apoptotic substrate cleaning in the testis and in the ovaries.

It has been established that the phagosomes formed downstream of MERTK activation in RPE, Sertoli and GC contained several autophagy markers, including lipidated LC3 protein [21,44,143]. These unconventional phagosomes were produced following the activation of LC3-associated phagocytosis (LAP), a “hybrid” process in which autophagy proteins supported phagocytosis machinery. Exploitation of the autophagy component increases the efficiency of phagocytosis, providing rapid lysosomal fusion and cargo degradation. Mice that are deficient for LAP, but not for canonical autophagy, accumulate apoptotic bodies within the cytosol of phagocytic cells [144]. The unconventional phagosomes containing LC3 proteins were termed LAPosomes. In contrast to autophagic vacuoles, the LAPosomes are single-membrane vesicles coated with lipidated LC3 protein [144].

LAP is a separate process, which strongly differs from both phagocytosis and autophagy. A major difference between LAP and classical phagocytosis is the accumulation of the lipidated form of the LC3 protein (LC3-II) during the intracellular management of apoptotic substrates. In macrophages, LAP is triggered by the engulfment of pathogens, immune complexes, and dying cells via engagement of Toll-like receptors (*TLRs),* Fc receptors (FcRs), and phosphatidylserine receptors (PtdSer-Rs) [145]. LAP-specific genes include *ATG5*, autophagy-related gene 7 (*ATG7*), *Beclin1, Rubicon* (RUN domain and the cysteine-rich domain containing Beclin 1-interacting protein) and *NOX2* (NADPH oxidase-2) [146,147]. The protein Rubicon acts as a molecular switch between the repression of autophagy and the activation of LAP [146]. While using several autophagy proteins, LAP is independent of the autophagy preinitiation complex (ATG13-ULK1 (Unc-51 like autophagy activating kinase 1)-RB1CC1/FIP200 (RB1 inducible coiled-coil 1)-ATG101) [143,147,148]. In macrophages and in retinal pigmented epithelial cells, autophagy components ATG5, ATG7, ATG3, ATG12, ATG16L1 and a Beclin1-PIK3C3 (Phosphatidylinositol 3-Kinase Catalytic Subunit Type 3)/VPS34 (vacuolar protein sorting) complex lacking ATG14 are engaged in the lipidation of LC3 protein and its recruitment to the phagosome. LAP also requires Rubicon for proper function [146,149]. During LAP, Rubicon is recruited to the LAPosome and is required for the activity of a PI3KC3 complex containing Beclin-1, UV radiation resistance-associated gene protein (UVRAG), and VPS34, but lacking the canonical autophagy components ATG14 and activating molecule in BECN1-regulated autophagy protein 1 (Ambra1). LC3, Beclin1, ATG5 and Rubicon proteins have been detected in LAPosomes from different types of phagocytic cells (blood macrophages, RPE cells, GC) that use LAP [21,44,144,146,150,151,152]. LC3, Beclin1, ATG12 and Rubicon proteins have been detected in LAPosomes formed by the Sertoli cells [18].

## 10. LAP Is a Degradation Process Different from Both Phagocytosis and Autophagy

**Figure 2:**
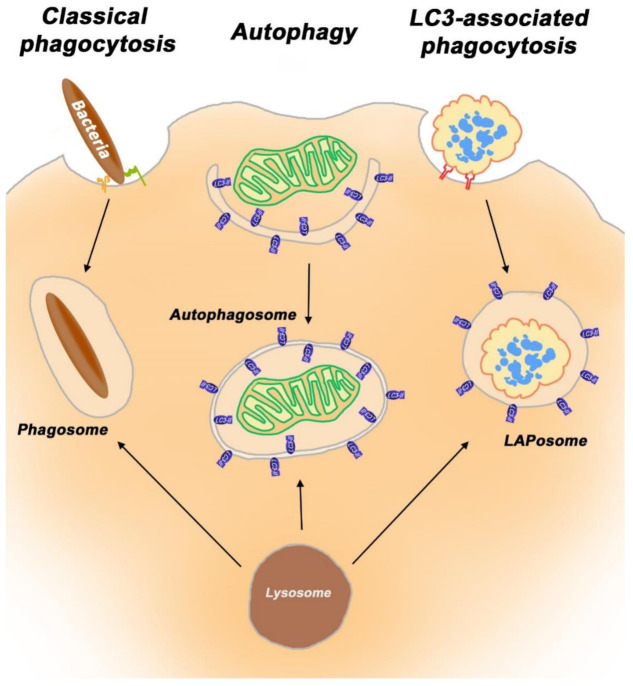
Phagocytosis, autophagy and LAP are functionally and mechanistically distinct degradative pathways.

TAM RTKs have been shown to trigger LAP in professional phagocytes after binding to PS on apoptotic substrates through bridging molecules such as GAS6 or ProS1. Alternatively, LAP is initiated upon the engulfment of apoptotic cells through T cell/transmembrane, immunoglobulin, and mucin (TIM)-1,3,4 receptors, or stabilin-1, stabilin-2 and brain-specific angiogenesis inhibitor (BAI1) receptors. LAP is also engaged by TLR receptors 1/2, TLR2/6 and TLR4 (see below) [144]. The presence of LC3 proteins distinguishes LAPosomes from single membrane phagosome vesicles [144]. LAP is also often termed as non-canonical autophagy [153] (Figure 2).

A major difference between LAP and conventional autophagy is the very short time taken for the accumulation of a lipidated form of LC3. Indeed, the lipidation of LC3 protein can be detected 15 min after the loading of an apoptotic substrate to a phagocyte cell [21,24,143]. Furthermore, 30 min after the binding of an apoptotic substrate to a plasma membrane, the LAPosome fuses with lysosomes, triggering a degradation process, as shown for RPE cells [154]. Such a high rate of LAP explains the interesting phenomenon regarding the absence of apoptotic germ cells from the sections of seminiferous tubules. Indeed, in the seminiferous tubules, germ cell death undergoes cyclic variations depending on the stage of the seminiferous epithelium cycle [19]. Nevertheless, neither massive presence nor cyclic variation of died germ cells in tissue sections and within the Sertoli cell cytoplasm has been detected [155,156]. To explain this fact, it has been hypothesized that the phagocytic elimination of apoptotic germ cells in the seminiferous tubules is a special, extremely fast process, which was unknown at that time [156]. Nowadays, the involvement of LAP in apoptotic germ cell cleaning is well documented [18]. Of note is that other substrates for removal by Sertoli cells (i.e., spermatid’s RB) seem to be processed through conventional phagocytosis machinery because their management does not involve LC3 recruitment [44].

**Figure 3:**
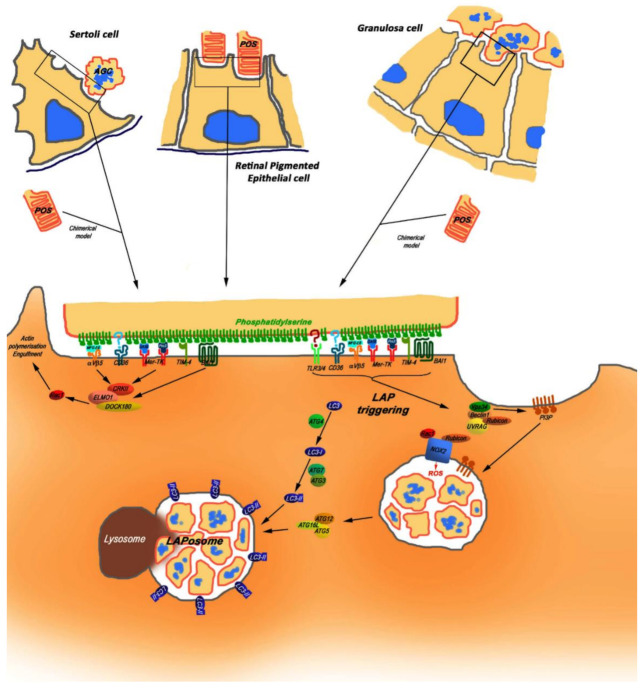
Elimination of apoptotic substrates in blood-separated retina, testis and ovaries. In each organ, specific non-professional phagocyte cells are in charge of the elimination of apoptotic substrates: Sertoli cells for germ cells in the testis, retinal pigmented epithelium cells for POS in the retina, and granulosa cells for apoptotic oocytes in ovarian follicles. In each case, and even for granulosa cells or Sertoli cells challenged with chimerical substrates (POS), LAP is triggered through the activation of different receptors recognizing specific molecules on the surface of apoptotic substrates. Among those receptors, MERTK plays a central role, allowing both the triggering of actin reorganization through Rho-GTPases, leading to engulfment of substrates, and assembling complexes of proteins accomplishing LAP. Abbreviations: LAP—LC3-associated phagocytosis; POS—photoreceptor outer segments, RB –spermatid’s residual bodies; AO—Apoptotic Oocyte; AGC—Apoptotic Germ Cell.

LAP and autophagy share many cellular resources, using common mediators such as ATG5, ATG7, LC3 and BECN1, and compete for lysosomes to provide cargo degradation [149]. In rodent RPE under physiological conditions, Mertk-MERTK-mediated autophagy-assisted phagocytosis and autophagy processes reach their peak at different times of day. This avoids the competition for lysosomal resources in vivo [149] and suggests a balance between autophagy and LAP in non-professional phagocytes. Indeed, starvation-induced autophagy impairs the degradation of POS [143,149]. In vitro study using a chimerical phagocytosis model, in which ovarian GC were used as non-professional phagocytes and retinal POS as apoptotic-like substrates, showed that the induction of autophagy influences the uptake of POS [21]. Indeed, the induction of autophagy by rapamycin treatment in GC dramatically decreased the number of ingested POS, while the number of bound ones presented no variation. Remarkably, in contrast to large pieces of bound POS, the POS ingested by GC were fragmented into small particles. The latter were almost absent from rapamycin-treated GC, suggesting that autophagy induction influences the processes of MERTK-mediating ingestion and/or fragmentation of apoptotic substrates by non-professional phagocytes. The role of the Rubicon protein providing the switch between autophagy and LAP seems likely to support such a mechanism [146] (Figure 3).

A recent study [154] using human pluripotent stem cell-derived RPE cells shed light on the role of MERTK in the fragmentation of POS. Indeed, in RPE cells, phagocytosis starts with the capture and ensheathment of POS by apical processes of RPE. The phagosomes/LAPosomes then ascend in the processes toward the RPE soma [157]. Ensheathment was stimulated by MERTK ligands, GAS6 and PROS1, but not by αVβ5 integrin receptor ligands, MFG-E8 and vitronectin. Remarkably, the ensheathment participates in fragmenting POS before their internalization. The authors suggest that this is necessary to ‘‘bite-off’’ POS fragments as eatable-sized portions from the photoreceptors, and that MERTK activation is required for ensheathment-mediated fragmentation of POS before internalization [154]. Moreover, the knocking down of MERTK in RPE resulted in complete failure of the ensheathment, fragmentation and internalization of POS, thereby leading to vision loss in patients and animal models. The rescue of MERTK expression in retinitis pigmentosa (RP38) patient RPE counteracted these defects [154]. The molecular mechanism downstream of MERTK activation which leads to fragmentation of apoptotic substrates remains to be uncovered. Knowledge of this mechanism would shed light onto the mode of fragmentation of large substrates, such as apoptotic germ cells or apoptotic oocytes, before their cleaning by non-professional phagocytes from testis and ovaries.

## 11. Klotho Is a Regulator of MERTK Expression

Because TAM tyrosine kinases are implicated in a variety of cellular metabolic pathways, the issue of regulation of their activities is of particular importance in several pathological states such as cancer and others. Therefore, different pharmacological modulators of TAM receptors have been elaborated [158]. Along with natural the TAM ligands listed above, these molecules can be used to target TAM receptors in different tissues.

To date, current knowledge on TAM receptor expression in human cells remains partial [159]. In the context of the present review, of special interest are the data on the regulation of MERTK expression by putative anti-aging gene Klotho (*Kl*) [160,161]. The Klotho (*Kl*) gene was first described in 1997 [160]. The study demonstrated that *Kl*-null mice displayed phenotypes resembling human premature-aging syndromes. Klotho is a transmembrane protein, which can be cleaved and shed, as well as act as a circulating hormone [160]. Klotho can be isolated from bodily fluids, including blood, urine and cerebro-spinal fluid (CSF) [162].

Studies of the *Kl−/−* mice phenotype supported the importance of TAM family receptors for the maintenance of tissue homeostasis in the retina, the testis and the ovaries. In retinal health and RPE physiology, Klotho was shown to play a key role [161]. Studies on the retinal *Kl−/−* mice phenotype revealed retinal degeneration associated with RPE abnormalities. Phagocytosis of POS by RPE was dramatically impaired, so that no phagosomes were detected in RPE of *Kl−/−* mice [161]. Further studies showed that the Klotho protein was expressed in cultured human RPE, taking part in the regulation of melanogenesis. Moreover, recombinant Klotho proteins increased phagocytosis in cultured RPE by inducing gene expression of MERTK/Axl/Tyro3 receptors. These effects of Klotho are mediated through cAMP-PKA-dependent phosphorylation of transcription factor CREB [161]. Of interest is that the qRT-PCR study of retinas from young and old mice revealed a significant decrease of expression of Klotho and Mer/Axl/Tyro3 in old mice compared to young mice. This is in good agreement with the age-dependent decrease of phagocytic activity of RPE, increased lipofuscin content and the loss of vision in elderly individuals and animal models [163,164].

Remarkably, *Kl−/−* mice also manifest severe degeneration of reproductive organs in both males and females [160]. In normal human testes, Klotho is expressed by somatic Sertoli cells [165], while in mice, Klotho expression is mainly confined to germ cells [166]. Of note is that the ovaries of *Kl−/−* mice manifested a cessation of follicular maturation at the pre-antral stage, and the presence of numerous atretic ovarian follicles—a feature of human polycystic ovarian syndrome (PCOS) [167]. As recently shown in humans, PCOS is associated with abnormal Klotho signaling [168]. In human ovaries, Klotho is expressed by GC [169]. Furthermore, inhibition of Klotho expression in ovarian GC by miR-15b induces premature ovarian failure (POF) syndrome [170], which is heralded by the abnormal depletion of ovarian reserve. Moreover, in both GC and serum derived from women with diminished ovarian reserve, Klotho expressions were significantly lower compared to normal individuals [170]. Of note is that both PCOS and POF are associated with the presence of autoantibodies against ovarian antigens [171,172], indicating the failure of timely and complete removal of apoptotic substrates from the ovaries. Thus, the data from the Klotho phenotype in ovaries are in agreement with the role of MERTK-mediated cleaning of apoptotic substrates by GC.

## 12. TAM Receptors as Negative Regulators of Inflammation: Two Possible Explanations

A study on triple mutant *TAM−/−* mice revealed severe consequences in the immune system, resembling the pathogenesis of systemic lupus erythematosus (SLE) [173]. This was manifested by a high titer of circulating antibodies to multiple autoantigens, including double-stranded DNA, a variety of plasma membrane phospholipids and collagen. In addition to developing autoimmunity, TAM deficient mice are hyper-sensitive to endotoxins, as demonstrated by the production of pro-inflammatory cytokines; for example, *Mer−/−* mice die from a dose of lipopolysaccaride (LPS) that is non-lethal in WT control [174,175].

Two possible explanations of this phenomenon have been proposed, the first of them is based on the involvement of TAM receptors in the LAP-mediated mechanism of apoptotic substrate elimination. Indeed, the similar SLE-like phenotype was described for mice lacking one or several components of the LAP pathway, triggered (among others) by the activation of TAM receptors [176,177]. Remarkably, dying cells, injected into LAP-deficient animals, are ingested by immune cells, but not efficiently degraded, and trigger acute elevation of pro-inflammatory cytokines. Repeated injections of apoptotic cells into LAP-deficient animals accelerated SLA-like diseases, including increased serum levels of autoantibodies [176].

A study using dystrophic RCS rat model with Mertk mutation showed that retinal autoantibodies were generated in response to antigenic material released from dying photoreceptor cells during retinal degeneration [178]. The excess of apoptotic germ cells, impairment of the blood-testis barrier, macrophage and lymphocyte infiltration, along with the emergence of autoantibodies against germ cell antigens, are also the hallmarks of seminiferous tubules from *TAM−/−* mice [179,180,181].

The second explanation regards the TAM-mediated inhibition of the TLRs-induced inflammatory cascade. The TLR family plays an essential role in activating signal transduction pathways leading to the killing and clearance of pathogens [182]. TLRs are expressed by immune cells, such as lymphocytes, dendritic cells and macrophages, and non-immune cells such as epithelia cells of many tissues. Different members of the TLR family are expressed in RPE, Sertoli cells and GC [183,184,185]. Activation of TLRs elicits host defense factors responsible for local inflammation, the recruitment of immune cells and the secretion of cytokines that modulate innate and adaptive immune responses [186]. Failure of the TLR fine tuning causes their unrestrained activation, generating an inflamed environment promoting autoimmunity [187]. Therefore, negative regulation of TLR signaling is tightly controlled on multiple levels [188], including those of degradation of signal proteins, mediated by the suppressor of cytokine signaling (SOCS). Evidence indicating that TRLs are the subjects of negative TAM regulation, mediated by SOCS, was firstly presented for professional phagocytes (dendritic cells) [10], then for testis Sertoli cells [179].

A detailed study on the role of TAM receptors in local inflammatory status in blood-separated tissues has been performed for TAM−/− triple mutant testis. Careful examination of pro-inflammatory cytokine levels in testicular extracts from TAM−/− mutant mice showed the increase of tumor necrosis factor α (TNF-α), interleukins (IL) IL-1β, IL-6 and I type of interferons (IFN) proteins compared to WT controls [179,180,181]. A further study of the mechanism underlying TAM-mediated inhibition of TLR signaling has been carried out using the cultured primary Sertoli cells from triple TAM−/− phenotype [179]. Thus, it has been established that stimulation of TLR3 by poly polyinosinic:polycytidylic acid (I:C) in the Sertoli cell resulted in nuclear factor kappa-light-chain-enhancer of activated B cells (NF-κB) activation and translocation from cytoplasm to the nucleus after the phosphorylation of its p65 subunit. Compared to WT animals, TAM−/− Sertoli cells exhibited an elevated level of phosphorylated p65. Then, incubation with GAS6 significantly decreased poly (I:C)-induced p65 phosphorylation in control Sertoli cells, but not in TAM−/− ones. As for other cell types, in Sertoli cells, TLR3 triggers the TIR domain-containing adapter-inducing interferon β (TRIF)-dependent pathway that leads to activation of IRF3 via its phosphorylation [179,180,181]. Pretreatment with TAM ligand GAS6 significantly inhibited interferon regulatory factor 3 (IRF3) phosphorylation in Sertoli cells from the WT control, but not from TAM−/− mice.

Further studies by the same authors demonstrated that TAM modulated inflammatory cytokine production by Sertoli cells [179,180,181]. Thus, upon the stimulation by poly (I:C), TAM*−/−* mice upregulated TLR3-driven inflammatory cytokines IL-1β, IL-6 and TNF-α, as well as the production of IFN-α and IFN-β compared to control animals. Moreover, TAM ligand GAS6 inhibited poly (I:C)-induced cytokine production in the WT control, but not in TAM*−/−* Sertoli cells [179,180,181]. Finally, in the Sertoli cells, the negative mechanism of TLRs modulation by TAM relies upon the expression of the suppressor of cytokine signalling (SOCSs) factors. Both SOSC1 and SOSC3 factors that mediate TAM inhibition of TLR signaling in professional phagocytes [10] were decreased in Sertoli cells from TAM*−/−* mice and did not exhibit any response to GAS6 stimulation. On the contrary, in control Sertoli cells, the expression of both supressor molecules enhanced by up to 60-fold in the presence of poly (I:C) and TAM ligand. As expected, GAS6 induced phosphorylation of transcription factor signal transducer and activator of transcription 1 protein (STAT1) that controls SOCS expression in WT, but not in TAM*−/−* mice [10]. Because SOCSs also inhibit the TLR-triggered myeloid differentiation protein 88 (MyD88) pathway, activated upon the recruitment of all TLRs except TLR3, the GAS6-induced effect on TLR signaling was assessed using TLR4 ligand LPS. Of note is that in the case of TLRs, the signaling molecules MyD88 and TRIF was dispensable for LAP [182]. Obviously, LPS-induced proinflammatory cytokine induction was significantly suppressed by GAS6 [179]. Collectively, the data suggest that TAM receptors inhibit both the TLR-triggered MyD88-dependent and TRIF-dependent pathways in Sertoli cells.

It should be noted that the cytokines exert direct effects on active endocrine tissues and are produced by them in noteworthy concentrations [183,184]. Thus, under physiological conditions, TNF- α, IL-1α, IL-1β and IL-6 are continuously expressed in the testis, without inducing any inflammation, but regulating spermatogenesis on different levels [185,189,190,191,192,193,194,195,196,197]. In the ovary, cytokines TNF-α, interleukins IL-1β -6, -8 and others [198,199,200] promote follicular growth, steroidogenesis, recruitment and activation of leukocytes necessary for ovulation and tissue remodeling during ovulation, luteinization and luteolysis [198,199,200,201]. When upregulated, they negatively affect spermatogenesis and oogenesis, leading to infertility inducing germ cell apoptosis, impairment of blood barriers, immune cell infiltration and the production of autoantibodies [202,203,204,205,206].

To our knowledge, no such detailed study on MERTK-mediated inhibition of TLR signaling in blood-separated retina and ovaries is available to date. MERTK was shown to be important for the inhibition of inflammation in macrophages [207] and macrophage-like cell lines [208] by driving the downregulation of LPS-induced production of TNF-α and IL-6, by triggering PI3K/protein kinase B (AKT) and NF-kB pathways. In M2c anti-inflammatory macrophages, the Mer/Gas6 axis can prevent the release of proinflammatory cytokines and can induce the expression of anti-inflammatory mediators [209,210]. In degenerative RCS rat retinas (mutation of MERTK), increased pro-inflammatory cytokines and activation of microglia were detected starting from early stages of retinal degeneration, supporting the role of MERTK in the regulation of the TLR-mediated inflammatory response [211,212]. Increased inflammatory status is also associated with ARMD—a leading cause of vision loss in elderly individuals. The decline of MERTK-mediated phagocytic activity of RPE is currently considered one of the major factors contributing to this multifactorial pathology [213,214].

Interestingly, alike Sertoli cells and GC [215,216,217], TLR3 and TLR4 are also expressed at the highest levels in RPE [218]. As suggested, TLR3 might contribute to the clearance of degenerating photoreceptor cells during pathological states in the retina. In these cases, TLRs are thought to be activated by RNAs released from degraded photoreceptors [219]. However, as with other cell types [220], activation of TLR3 in RPE cells triggers their own apoptosis, thereby enhancing a deleterious effect for the retina. To date, it is not clear whether TLR3 contributes to POS phagocytosis in non-pathological situations. On the contrary, in vitro, TLR4 facilitates the phagocytosis of POS by generating transmembrane metabolic and calcium signals that contribute to POS ingestion. In the RPE-retina interface, TLR4 clusters at the sites of contact of POS with MERTK, CD36 and integrins, forming the supra-molecular complex, where each receptor plays a particular role to provide substrate clearance. According to the authors, the role of CD36 is to be a participatory recognition molecule, and the role of MERTK is to be a phagocytosis signaling molecule, while TLR4 is an activating stimulus, specific to the molecular pattern of POS [221]. To what extent such a model can explain the regulatory axis between MERTK-mediated phagocytosis and TLR-mediated inflammation is to be discussed, taking into consideration that, in macrophages, TLR4 triggers the non-inflammatory LAP mechanism [182].

## 13. Conclusions

Timely and efficient elimination of apoptotic substrates, continuously produced during one’s lifespan, is a vital need for all tissues of the body. In blood-separated tissues, local non-professional phagocytes cells combine various mechanisms allowing the maintainance of tissue homeostasis and the avoidance of acute inflammation, tissue disorganization and functional ablation. A deeper knowledge of regulatory mechanisms supporting tight cooperation between TAM RTKs and TLR receptors, autophagy-related proteins and LAP will contribute to providing important basic resources to counteract several vision and reproduction pathologies.

## Author Contributions

M.G.Y. and B.J.; methodology, M.G.Y., C.R., N.B; software, N.B., A.D.R.; validation, M.G.Y., N.B., C.R., B.J.; formal analysis M.G.Y.; investigation M.G.Y.; resources C.R., A.D.R., B.J.; data curation M.G.Y.; writing—original draft preparation, M.G.Y. and N.B.; writing—review and editing, M.G.Y. and N.B.; visualization, M.G.Y. and N.B.; supervision, C.R. and B.J.; project administration, B.J.; funding acquisition, C.R., A.D.R, N.B., B.J. All authors have read and agreed to the published version of the manuscript.

## Data Availability

The data presented in this study are available on request from the corresponding author.
